# Microbiota and Obesity: Where Are We Now?

**DOI:** 10.3390/biology9120415

**Published:** 2020-11-25

**Authors:** Andrea Ballini, Salvatore Scacco, Mariarosaria Boccellino, Luigi Santacroce, Roberto Arrigoni

**Affiliations:** 1 Department of Biosciences, Biotechnologies and Biopharmaceutics, Campus Universitario, University of Bari “Aldo Moro”, 70125 Bari, Italy; andrea.ballini@uniba.it; 2 Department of Basic Medical Sciences, Neurosciences and Sense Organs, University of Bari “Aldo Moro”, 70121 Bari, Italy; 3 Department of Precision Medicine, University of Campania “Luigi Vanvitelli”, 80138 Naples, Italy; 4 Microbiology and Virology Laboratory, Ionian Department, Policlinico University Hospital, University of Bari “Aldo Moro”, 70124 Bari, Italy; luigi.santacroce@uniba.it; 5 CNR Institute of Biomembranes, Bioenergetics and Molecular Biotechnologies (IBIOM), 70124 Bari, Italy

**Keywords:** microbiota, obesity-related metabolic diseases, dysbiosis, probiotics and prebiotics, fecal microbiota transplantation, clinical biochemistry and clinical molecular biology, immune system, clinical microbiology, impact of nutrition and physical exercise activities in health

## Abstract

**Simple Summary:**

Emerging new data reported in the international scientific literature show that specific alterations in the human gut microbiota are characteristic in obesity and obesity-related metabolic diseases. Obesity is conditioned by a multitude of factors, and the microbiota is certainly an important player. The analysis of the data obtained from experimental studies allow us to hypothesize that changes in the composition of the microbiota may be the cause, and not simply the consequence, of alterations in human metabolism. Clinical trials on wide samples that investigate the role of diet-induced modulation of the gut microbiota on the host metabolism are needed to understand the interactions at the molecular level for the observed correlations between metabolism and microbiota changes.

**Abstract:**

Genetic and environmental factors are underlying causes of obesity and other metabolic diseases, so it is therefore difficult to find suitable and effective medical treatments. However, without a doubt, the gut microbiota—and also the bacteria present in the oral cavity—act as key factors in the development of these pathologies, yet the mechanisms have not been fully described. Certainly, a more detailed knowledge of the structure of the microbiota—composition, intra- and inter-species relationships, metabolic functions—could be of great help in counteracting the onset of obesity. Identifying key bacterial species will allow us to create a database of “healthy” bacteria, making it possible to manipulate the bacterial community according to metabolic and clinical needs. Targeting gut microbiota in clinical care as treatment for obesity and health-related complications—even just for weight loss has become a real possibility. In this topical review we provide an overview of the role of the microbiota on host energy homeostasis and obesity-related metabolic diseases, therefore addressing the therapeutic potential of novel and existing strategies (impact of nutrition/dietary modulation, and fecal microbiota transplantation) in the treatment of metabolic disease.

## 1. Introduction

In recent years we are witnessing an epidemic increase in obese people worldwide: according to the most recent data, it has been estimated that more than 2 billion people are overweight or obese worldwide [1], and in 2030, 42% of the adult US population are expected to be obese [2]. Adult obesity is associated with an impaired quality of life and with a myriad of health problems, including metabolic syndrome, diabetes, cancer, and respiratory and cardiovascular problems, and predisposition to the onset of metabolic syndrome [3,4].

The microbiota is a crucial factor for human metabolism, ultimately influencing our health or conversely, favoring the onset and evolution of various pathologies. It is widely accepted that the microbiota, including oral microbiota, is related to obesity development [5]. The correlation between oral microbiota and obesity has been reported in numerous scientific papers, starting from different bacteria composition [6] that can lead to an altered taste perception [7], favoring the consumption of so-called comfort food, usually caloric dense.

The undigested component of every food we eat becomes a food source for our microbiota, which in turn gives us back other metabolites, which are not only important for energy harvesting, but also for their role in the regulatory and signaling pathways. It is becoming increasingly evident that the microbiome has a fundamental role in the appearance and development of various pathologies, both chronic and infectious. It is therefore quite logical to consider the microbial community as a potential target of clinical strategies for identifying and treating various pathological situations. Despite a considerable amount of studies on the microbiota and obesity connection, a clear explanation at the molecular level remains uncertain [8]. Promising molecular connections have been recently demonstrated in mice, yet the multiple factors underlying human obesity and related-metabolic dysfunction (including genetics/epigenetics and lifestyle) make it difficult to demonstrate an independent role for gut dysbiosis. Biological science is perfectly capable of characterizing all the individual aspects of the microbial community (e.g., composition, functions, metabolites produced, relationship between species) but it is not able to clearly define the contribution of each of these aspects to the possible development of metabolic alterations and any pathologies, and therefore possible applications for therapeutic purposes.

## 2. The Gut Microbiota

It is well established in the scientific literature that there are substantial differences, both in composition and function, in the microbiota of obese versus healthy individuals [9]; unquestionably eating habits—for example, the Western diet, or a very restrictive diet—can contribute to altering these parameters, increasing the metabolic stress, which in turn contributes to the development of obesity [10]. The microbiota dysbiosis in obesity is characterized by a substantial reduction in diversity in the microbial community, shown, for example, as an increased ratio of *Firmicutes* vs. *Bacteroidetes* [11]. Interestingly, if the subjects have a weight loss, the altered proportion can be reversed [11,12,13]. These compositional differences are reflected in major alterations of the microbiota metabolic functionality: in obese individual, there is a substantial increase in short chain fatty acid (SCFA) producers (*Actinobacteria*), and pathogenic *Proteobacteria*, suggesting a possible role of this kind of bacteria in obesity metabolic changes [14].

Regarding the microbiome composition, metagenomics analysis has shown that 40% of the gut microbial gene pool is shared among all individuals around the world, thus supporting the existence of a core microbiome (Table 1). [15]. Although, due to the generation of different outcomes depending on the algorithm used, this concept is debatable, the notion of a microbiota fingerprint, a microbial stability over time—regardless of all the variation possibilities (i.e., age, lifestyle, gender, diurnal changes, sedentary, physical activity, etc.)—enables the conservation of several important functional pathways including energy metabolism [16,17].

Projects such as the Human Microbiome Project and American Gut Project can be very helpful in the gut microbiome analysis, helping us to elucidate the roles and the relationships of microbes in health and disease states.

Gut microbiota alterations have been found not only in obesity but in multiple pathologies, ranging from intestinal bowel syndrome (IBS), type 2 diabetes (T2D) [18,19,20,21,22,23], and cardiovascular diseases [24] to Parkinson [25]. The real problem is to give an absolute and causal value to the variations in the composition; more recent studies have found different variations, hence excluding a clear taxonomic signature for the development of obesity [26].

The molecular mechanisms that connect the gut microbiota with obesity are still elusive, but include energy harvesting from food, gut barrier permeability and immune system functionality, and production of specific metabolites that influence energy metabolism and signaling pathway, producing visible effects on the human metabolism in its entirety.

It is impossible to talk about obesity and the microbiota without bringing up dietary habits. The nutritional modulation of gut microbiota can influence total energy intake, nutrient absorption, transport and storage, which are reflected downstream on the general host metabolism, ultimately benefiting your health or instead promoting weight gain. For example, people who routinely consume a diet high in fiber and polyphenols, have a greater diversity of microbiota, which can be defined as substantially healthier, whereas lower richness was found in Western diet consumers [27].

Drug and antibiotic treatments are another notable factor affecting gut microbiota composition. The microbiota is deeply influenced by chemotherapy, in its composition and functionality, which is compromised, favoring the appearance of opportunistic infections [28]. Metformin, a well-known drug used for diabetic treatment, is able to act directly on microbiota [29,30].

However, it should be emphasized that, the simple observation of changes in the relationships between the different bacterial phyla as a causal factor of obesity has obvious scientific limitations. It could be a consequence rather than the cause of obesity, and moreover, it does not sufficiently take into account the role changes that can occur within species or strains, even with horizontal gene transfer. High species diversity does not equally correlate with gene content/diversity and even good health. Metagenomics studies are clearly highlighting how the metabolic potential of the microbiota is much more complex than the simple taxonomic observation of which species are present. It would therefore be more appropriate to try to fully understand the molecular mechanisms underlying the association of obesity and dysbiosis of the gut microbiota.

## 3. Metabolic Function and Interaction with Host Metabolism

The microbiota interaction with our metabolism is larger than originally thought: with mutual influences with diet lifestyle and genetic background the bacterial community can affect—directly or indirectly—energy harvesting, immune function, hormones secretion, gut barrier permeability and even mood and nervous system. 

**Figure 1:**
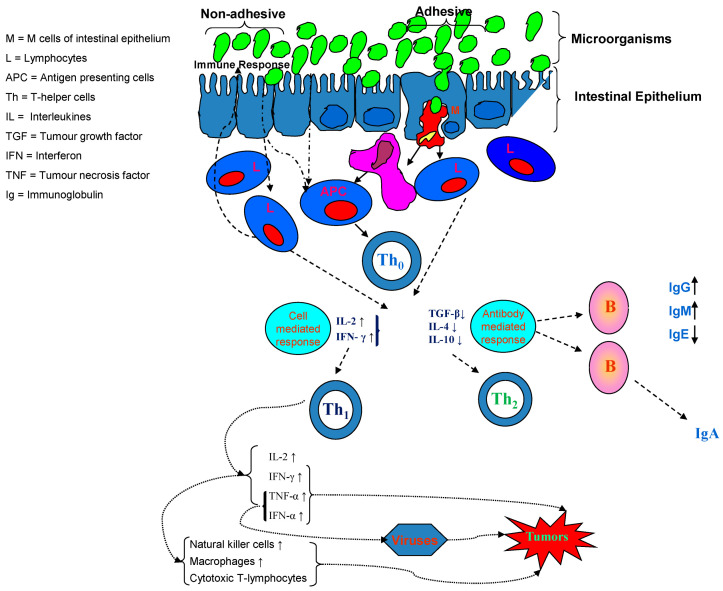
Modulating the gut microbiome: the microbiota maintains intestinal integrity with gut associated lymphatic tissue T cell activation and differentiation.

The microbiota plays a role in maintaining the gut barrier function, an important and strategic defense, critical for our health. The barrier prevents the entrance of potentially pathogenic bacteria and antigens, and eventual competition for nutrients and adhesion, thus avoiding an inflammatory state and possible recruiting of cells of the immune system [30]. The microbiota nucleotide-binding oligomerization domain-containing protein (NOD) 1 and 2 help the lymphoid tissue in preventing inflammation and promote epithelial cells regeneration [31]. Concerning nod-like receptor containing N-terminal pyrin domain (NLRP) NLRP1, NLRP3, and NLRC4 inflammasomes, it has been shown that upstream signals for these inflammasomes are relatively well-defined and can be both microbial- and/or host-derived (Figure 1) [32]. 

In particular, the NLRP3 inflammasome is unique in that it responds to a diverse array of stimuli, both microbial and non-microbial, such as bacterial pore-forming toxins, particulate matter, oxidized mitochondrial DNA, and extracellular ATP [33].

More recently, the NLR family member NLRP6 has been considered to be capable of regulating goblet cell function through autophagy pathways, and also of promoting Muc2 secretion in response to bacterial signals, both of which are important for maintaining an intact mucus layer that provides an effective barrier against bacterial-driven inflammation [34]. 

The microbiota and its metabolites are crucial actors in shaping the gut barrier structurally and functionally, by the production of amino acids, antimicrobial compounds and phenolic derivatives [33].

The bile acids metabolism is heavily influenced by the gut microbiota composition. Bile salt hydrolases (BSHs), a key enzyme for deconjugation of converting primary bile acids into secondary bile acids, is produced by distinct bacteria genera (*Bacteroides*, *Bifidobacterium*, *Clostridium*, *Lactobacillus*). Other bile acids reactions (7alpha-dehydroxylation, esterification and oxidation/epimerization) also require the gut microbiota as well, ultimately controlling deoxycholic acid levels, which in turn inhibits its receptors, mainly farnesoid X receptor (FXR) and Takeda G protein-coupled receptor 5 (TGR5) [35]. FXR and TGR5 are fundamental in modulating the human global metabolism: FXR regulates fatty acid uptake, synthesis and oxidation in liver; TGR5—also with FXR cooperation—promotes glucagon-like peptide-1 (GLP-1) secretion, leading to increased insulin secretion and reduced glucagon synthesis, regulating energy expenses and insulin sensitivity in skeletal muscle, and has an anti-inflammatory effect [36]. It is noteworthy that butyrate—whose levels are reduced in dysbiosis—can upregulate GLP-1R expression; supplementation of sodium butyrate is able to prevent simple steatosis progression to steatohepatitis in NAFLD patients [37].

Notably, diet also has an immediate and dramatic impact on the microbial community, both in healthy than obese individuals. Excess of dietary products such as seafood or fish can increase the gut bacteria production of trimethylamine (TMA), potentially involved in cardiovascular disease [38]. TMA and its product, trimethylamine N-oxide (TMAO), were identified by screening metabolites associated with cardiovascular disease (CVD), and TMA require gut bacteria for its formation [39]. After formation and absorption in the colon, TMA passes into the portal circulation, which directs blood into the liver, where it is oxidized to TMAO by flavin-containing mono-oxygenase 3 (FMO3) [37]. Oral antibiotics block the increase in TMAO that normally occurs after dietary challenge with either choline or carnitine, demonstrating that the generation of TMAO requires microbial bacteria [38]. Diet is the major cause of altering microbial structure in mice, more than genetic background [35]. It has been shown that the microbiota of people who follow a Western diet has very specific features, with a deeply changed structure, even without appreciable differences at the species or genes level [35]. Moreover, every dietary regime seems to have its own peculiar characteristics, differing from other diets. Experiments by Sonnenburg and colleagues [36] showed that diets unsuitable for the well-being of the microbiota destroy the microbial community, leading to the disappearance of species and strains intently and laboriously created throughout life, sometimes even over generations. Unfortunately, dietary changes (improvement) alone are not able to compensate for the loss of microbial species: only by using different techniques—especially fecal microbiota transplantation (FMT)—was it possible to restore the original microbiota richness in mice. Using these studies as a model to overcome bacterial loss and poor diversity, effective strategies could comprehend probiotics supplementation or FMT.

## 4. Obesity and Other Metabolic Pathologies

Microbiota dysbiosis is associated with several pathologies: the onset of obesity, metabolic syndrome, diabetes, intestinal bowel disease, liver diseases such as cirrhosis and NAFLD, and also with cancer; they have all shown correlations with changes in the gut microbiota [37].

What is the connection between obesity and microbiota? The pioneering experiments in the early 2000s on germ-free (GF) mice from Bäckhed and colleagues were instrumental in establishing the obesity–microbiota relationship [38]. After a microbiota transplant from the conventionally raised obese mice, the GF-mice showed an altered metabolism, becoming insulin resistant and consistently increasing in fat mass, even with a caloric restricted regimen [39]. The data clearly indicate the microbiota role in energy harvesting, even in a calorie-restricted regime, and notably that microbiota transplant is able to modify several metabolism characteristics in the recipient mice [39].

The relationship between microbiota imbalance and overweight and obesity may be due to many factors; various mechanisms have been hypothesized over the years: certainly, the microbiota metabolites seem to play a prominent role. SCFA represents about 10% of the energy resources for our metabolism [40]. In overweight and obesity individuals the SCFA levels are substantially higher—a probable consequence of the caloric excess—compared to non-obese individuals [38]. This is an extremely significant aspect, not only from a purely energetic point of view, but also for their potential role as signaling molecules, capable of producing functional changes in the host’s metabolism. For example, SCFAs are involved in glucose homeostasis. Furthermore, the anti-inflammatory action of SCFA—through histone deacetylases (HDACs) inhibition, Toll-like receptor and by stimulating the differentiation of T regulatory cells—should not be underestimated [40]. Hence, some of the metabolic consequences could be somewhat explained by the increased production of SCFAs observed [36]. SCFA are likely involved in glucose homeostasis—improving glucose sensitivity—and lipid metabolism through free fatty acid receptors (FFAR2/FFAR3), which leads to an activation of AMP-activated protein kinase. The metabolic consequences stimulate the activation of the hepatic gluconeogenesis and lipogenesis pathways [40], simultaneously inhibiting the fatty acid oxidation in muscles [41]. 

Increasing the permeability of the intestine enhances the uptake of LPS and leads to systemic inflammation [42]. Experiments on mice have demonstrated the direct connection between the presence of *Akkermansia muciniphila*, mucus layer and reduced intestine permeability, thus preventing the inflammation process [42]. 

In 2016, Udayappan et al. [43] showed that daily treatment for four weeks with *Eubacterium *hallii** L2–7 in severely insulin resistant *db/db* mice has no adverse effects and exerts beneficial effects on metabolism, potentially via alterations in butyrate formation and bile acid and metabolism.

In another study, mono-association of germfree C57BL/6J Enterobacter-induced obese mice with strain *Enterobacter cloacae B29* isolated from volunteer’s gut showed increased serum endotoxin load and aggravated inflammatory conditions [44]. The obesity-inducing capacity of this human-derived endotoxin producer in gnotobiotic mice suggests that it may causatively contribute to the development of obesity in its human host [44].

Gut bacteria can differentially regulate lipid metabolism. Indeed, products secreted by *Desulfovibrio* upregulate CD36 expression, whereas products produced by Clostridia can downregulate CD36 expression. Therefore, the loss of organisms that function to temper CD36 expression may lead to the inappropriate absorption of lipids, which can accumulate over time, leading to obesity and metabolic syndrome [45].

Truax et al. show that myeloid-expressed NLRP12 restrains high-fat diet-induced obesity and type 2 diabetes by attenuating TNF, IL-6, NF-kB, MAPK, M1 macrophage polarization and inflammasome activation in adipose tissue. This protective function of NLRP12 is microbiota dependent, and is associated with *Lachnospiraceae* and their metabolites, which mitigate obesity [46].

In addition to the role played in energy metabolism, SCFAs are important in the defense against pathogens at the level of the gut barrier. In experiments on mice, low levels of butyrate—due to a loss of *Clostridia*—could alter the oxygenation state in colonocytes, which in turn promote the infection of opportunistic bacteria [43]. Inflammatory signals from adipocytes, abundant in obese individuals, could compromise the gut barrier integrity and function, facilitating the translocation of pathogens and their proinflammatory molecules [44]. Hence, the obesity-related chronic low-grade inflammatory state could be facilitated by microbiota alterations [45]. 

Peroxisome proliferator-activated receptor gamma (PPARγ) could be involved in another plausible mechanism by triggering FIAF (fasting-induced adipose factor) activation, an enterocyte protein with an inhibitory effect on lipoprotein lipase (LPL), a key factor in lipid metabolism. The microbiota is also involved in the regulation of FIAF expression: a microbiota dysbiosis can suppress FIAF expression, leading an increased LPL activity and lipid accumulation in adipose tissue [47,48].

The link between obesity, metabolic disease and lower microbial diversity has been elucidated in several studies; this has been confirmed analyzing fecal microbiota from people of USA urban area, where fiber consumption is low and obesity percentage is high, compared to people of rural areas in Africa or South America [49]. Using metagenomics analysis, a TwinsUK cohort study—the largest adult twin registry and the most clinically detailed in the world—found that lower diversity was associated with greater abdominal adiposity [50].

In summary, less microbial diversity seems to be a consistent feature in obese individuals, even though the gut microbiota composition in them is not uniform. Certainly, those changes affect the microbiota metabolite production, with a marked effect on energy metabolism, on the functionality of the intestinal barrier and inflammatory problems. The altered production and metabolism of SCFA seems to be a recurring theme in the consequences provoked by microbiota dysbiosis; however, other bacteria metabolites are important in energy homeostasis, in immunoregulatory systems and defense and inflammation developing. 

Genetics, age, physical activity and many other factors have a substantial impact on the function of the intestinal flora, making it difficult to establish causal relationships on the role of the microbiota in the development of obesity [51]. In mice, physical activity promotes gut microbiota diversity [52]: low intensity exercise limits the transient stool time in the intestine—thereby reducing the risk of colon cancer, diverticulosis, and inflammatory bowel disease. In humans, gut microbial diversity is associated with cardiorespiratory fitness [53].

The ageing progression is correlated with a reduced diversity in the human microbiome, a plausible contributing cause of the frailty usually observed in older people [54]. Identifying the molecular modifications underlying these changes can be of great help to fight more effectively—perhaps even with the use of suitable probiotics—the consequences of aging.

**Figure 2:**
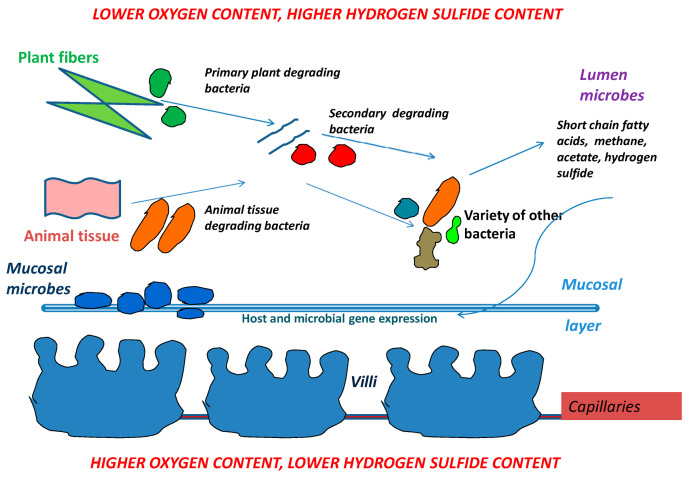
Simplified version of microbial community interactions. The intestinal wall is at the bottom, with villi and blood supply which brings oxygen to the intestinal wall. The top of the figure illustrates the mid-section/lumen of the intestine which has low oxygen supply but a higher supply of hydrogen sulfide from the sulfate reducing bacteria living in the lumen.

Animal and plant tissues from food are degraded in many steps by a variety of bacteria. Short chain fatty acids, methane and acetate are generated by bacteria involved in food breakdown. Those products then turn on or off both host genes and genes from other neighboring microbes. The mucosal layer covers the villi and also provides a home for the long term residence of mucosal microbes. When mucosal microbes populate the mucosal layer, they reside there for the lifetime of the host and rarely leave. In contrast, lumen microbe populations can change rapidly in quantity and type as changes occur (i.e., in food supply, food amount, pathogens) (Figure 2).

## 5. Food and Microbiota: Mutual Influences

There is a deep and intertwined relationship between the intestine and the central nervous system that regulates the appetite. Nutritional status, metabolic energy levels, secretion of peptides at the nervous and enteric level—the latter also dependent on the presence of food in the intestine—and hormonal signaling are all factors that regulate calorie intake [55]. It has already been shown that some bacterial strains are able to influence the synthesis of gut hormones, such as YY, GLP-1, leptin, and ghrelin, regulating appetite and satiety in the brain [56,57].

The gut–brain axis (GBA) is a bidirectional link between the central nervous system (CNS) and the enteric nervous system (ENS) of the body. It involves direct and indirect pathways between cognitive and emotional centers in the brain with peripheral intestinal functions. The GBA involves complex crosstalk between the endocrine hypothalamic–pituitary–adrenal axis (HPA), immune (cytokine and chemokines) and the autonomic nervous system (ANS) [57].

The GBA primarily combines the sympathetic and parasympathetic arms of the ANS, which drives both afferent and efferent neural signals between the gut and the brain, respectively. The HPA axis meanwhile coordinates adaptive responses against stress including activation of memory and emotional centers in the limbic system of the brain [58].

The neuro–immuno–endocrine mediators of the GBA allow the brain to influence intestinal function (immune cells, epithelial cells, enteric neurons, and smooth muscle cells). Moreover, the cells of the gastrointestinal (GI) tract are also under the influence of the gut microbiota and recent evidence suggests that there is an emerging concept whereby the microbiome plays an important role in the GBA structure [59,60].

Microbiota-derived metabolites, such as short-chain fatty acids, can bind to receptors on enteroendocrine cells, modifying the release of enteric hormones into the systemic circulation [58,59]. A diet high in non-digestible carbohydrates can enhance SCFA levels and gut hormone levels, in both animals and humans [58]. 

The role of the microbiota metabolite is very broad and not fully understood. A bidirectional crosstalk between the intestine, the enteric nervous system and the brain—mediated by the nervous system—does exist, and it is of the utmost importance in regulating our metabolism. It is noteworthy that the microbiota is capable of synthesizing metabolites, such as serotonin and γ-aminobutyric acid (GABA) [60], which are active in the central nervous system, that are involved in the control of appetite and thus indirectly in body weight regulation [61,62,63]. Acetate, an SCFA produced by intestinal bacteria, is able to act as in the hypothalamus by inhibiting the appetite stimulus; Byrne and colleagues demonstrated that intravenous acetate supplementation in rodents can influence anorexigenic signaling in the appetite center, inhibiting the appetite stimulus [61]. Interestingly, microbiota dysbiosis in obesity could enhance the acetate levels, promoting glucose-stimulated insulin secretion, increased ghrelin secretion, further increasing obesity [64]. 

In the psychiatric sciences disorders and obesity are often connected: after all, it is a common experience for somebody anxious or depressed to seek comfort in food, often selecting foods with high caloric intake [65]. Hence, the microbiota plays an important role with some neurological display, probably also because it acts as a link between the brain and our diet: experiments transplanting the microbiota from mice featuring psychological disorders also made the recipient mice depressed [66,67,68]. 

Many species of *Lactobacillus* and *Bifidobacterium* produce GABA, which is the main inhibitory neurotransmitter in the brain. In addition, *Candida*, *Escherichia*, and *Enterococcus* produce the neurotransmitter serotonin, while some *Bacillus* species have been shown to produce dopamine. Bacteria also produce SCFAs, such as butyric acid, propionic acid and acetic acid, that are able to stimulate sympathetic nervous system, mucosal serotonin release and thus influence the memory and learning process in the brain [63,64,65].

The symbiotic relationship between microbiota and host metabolism is well known. Mills and his colleagues in their work demonstrated that the first beneficiaries of oligosaccharides (HMO) present in the human milk are bacteria, and not the newborn. It was demonstrated that *Bifidobacterium infantis* is able to grow using HMO as an exclusive food source [69]. Hence, the interaction between human breast milk and *Bifidobacterium infantis* is similar to recruiting a new player—the bacterium itself—in the mother–newborn relationship.

Unfortunately to translate these multiple interactions in dietary indications beneficial to our health is extremely complex and difficult. To establish a direct link between a specific bacterial strain and modification of our metabolism is relatively an easy task; but to demonstrate such a specific connection with a probiotic is definitely challenging (or, with a prebiotic—which is even more complicated).

## 6. Dietary Interventions and Microbiota Modulation

Diet and microbiota are definitely linked in human health and disease. We now know with certainty how our lifestyle, and above all our eating habits, are able to produce profound changes in our intestinal flora, changing its composition, ecology and functionality [55]. There is increasing evidence that a diet rich in polyphenols—present mainly in foods and beverage of plant origin—can have an anti-oxidant and anti-obesity effect in humans [27,70]. A healthy and balanced diet, which also includes a correct daily consumption of calories—nowadays in the Western world a target to be pursued with difficulty—produces positive effects on our health, with different molecular mechanisms, some of which directly involve the microbiota [27].

Our microbiota and its salient characteristics—composition, functions, ecology—are influenced by numerous factors, some not modifiable (way of birth, genotype, age, ageing), but many instead modifiable by our behavior: physical activity, sedentary lifestyle, characteristics of the surrounding environment, temperature, etc. [71]. Surely our nutrition plays a fundamental role in shaping the microbiota: it is estimated that it affects up to 60% of the composition of our bacterial flora [72]: what we eat has a profound effect on our microbial community, shaping it according to needs (Table 2). 

It is evident that an excessively caloric diet, or too rich in fats, carbohydrates and/or proteins, such as the so-called Western diet, if constantly prolonged over time, determines decisive structural changes in the microbiota gut. Even what we do not eat—fiber, micronutrients, minerals—can significantly alter intestinal bacteria. A diet without dietary fiber—and therefore without entry of poorly digestible carbohydrates present in the fiber—usually leads to a reduced diversification of the bacterial flora [34,82].

These data have been confirmed by experiments conducted on mice on a diet high in fats and sugar—such as the classic Western dietary regimen which made mice fatten and modified the microbiota, altering the ratio between *Firmicutes* (highly capable of metabolizing simple carbohydrates) and *Bacteroidetes*, significantly reducing the number of the latter [83]. The importance of the eating habits is confirmed by the minimizing of the microbiota alteration whether mice are brought back to a diet that limits weight gain, emphasizing the close connection between microbiota metabolic functionality and nutrition.

One fundamental question arises from the food–microbiota interaction: is it possible to prevent disease through diet? The answer for the time being is still unclear; it should be maybe, but it must certainly be tailored for every single person.

### 6.1. Caloric Restriction 

Caloric restriction seems to be an important tool at our disposal: it was recently demonstrated that just one month of 40% CR improves insulin sensitivity, decreases body weight (BW) and white fat mass gain, without any discernible downside effects [84]. Analyzing the gut microbiota in these conditions showed a progressive change in the composition, perhaps supported by the improved insulin sensitivity and the less in adiposity. Interestingly CR-microbiota-transplanted mice—in the same dietary and caloric uptake condition—gained less weight in comparison with the controls. 

Life-long CR enhances the percentage of bacterial strains that are positively correlated with lifespan expectancy [85], and at systemic level the serum levels of the LPS-binding protein (LBP), are significantly lower. CR may affect blood LPS levels by negatively regulating its biosynthesis.

The microbiota alteration contributes significatively to many of the metabolic changes seen during CR: it is plausible that the lower concentration of SCFA found in mice during CR [86] could contribute to improve the glucose energy metabolism observed during CR. Besides the lower SCFA production—especially butyrate and propionate—may prevent the stimulation of adipocyte proliferation [87] and attenuating signaling of satiety [88].

### 6.2. Probiotics and Prebiotics

The scientific literature is literally inundated with many reports on the administration of prebiotics or probiotics to modify, nourish and cultivate the gut microbiota for health purposes. Probiotics are live microorganisms that are beneficial to the host, while prebiotics are biologically active substances capable of positively influencing the growth and the activity of intestinal flora, ultimately modifying various pathways in the host metabolism, including energy, regulatory and immune system. The real issue here is whether these effects are contradictory; it is still controversial whether there is a direct and causal connection between the use of prebiotics and changes in the microbiota or are just consequences of other changes already in place [89].

Prebiotics are defined as a group of nutrients directly utilized by gut microbiota; they promote the growth, development and function of bacterial strains that contribute to the well-being of both the microbial community itself and the human body [90,91,92,93,94] (Table 3). 

They have multiple effects modifying host metabolism: it is already known that orally ingested probiotic bacteria could improve atopic dermatitis symptomatology [95], modulate immunoregulatory cells via TLRs receptors, acting primarily on the regulation of carbohydrate metabolism [96]. However, the efficacy of the effect of prebiotics on the microbiota has many aspects to be taken into account, starting with the type of prebiotic administered, at the dose present in the food, and ending with the multiplicity of metabolic regulations in humans.

Gut microbiota is a key component to our health—the consumption of food containing probiotics has increased significantly, maybe hoping to treat—or ameliorate—a host of conditions. Some probiotics strains might look promising for treatments of Crohn’s disease [97]; still, none of the articles cited found substantial evidence to recommend their use [98].

Translating probiotic science into probiotic foods requires some scientific challenges: according to the latest research, variance in the personal microbiota and intestinal mucosa colonization can significantly alter the expected outcome of probiotic ingestion [99]. To support that probiotic-mediated changes in the microbiome confer health benefits for the host, more substantial evidence is needed: most of the studies on probiotics effect on gut microbiota changes have been made in animal models; clinical trials on humans are limited and sometimes contradictory [100]. Probiotics have been used to treat conditions associated with a disrupted microbiome, but, despite encouraging in vitro experiments [81], they have shown limited efficacy in vivo [101]. Furthermore, the risks of counterproductive effects of a probiotics administration can be underestimated: it can even slow down the restoration of the desired intestinal flora in human volunteers [102].

## 7. Therapeutics Strategies Targeting Microbiota 

It is very challenging to propose useful strategies regarding gut microbiota modifications and beneficial health consequences in humans. The real question is how to assess what is a healthy microbiome, and what bacteria strains it should be made of, taking into account our own personal uniqueness, although there are clearly communities at the family and class levels that have been identified as consistent with gut health [103].

Moreover, the most important question regarding the gut microbiota should be focused on which species are really metabolically active and therefore modulating the host’s metabolism, rather than worry about the relative abundances of the various species. It should be very useful as a diagnostic tool for the use of microbiome signatures as biomarkers for disease presence; it is well known that all metabolic diseases involve some type of microbiota dysbiosis—both as a cause and effect but it will not be an easy task.

Another line of research regarding the microbiota that could be beneficial for our health is the use (or promoting the production) of small molecules, anti-inflammatories, produced by members of the bacterial community for therapeutic purposes. Their ability to modulate host signaling pathways, and host physiology is a fascinating topic, yet remains to be elucidated. Intestinal bacteria metabolites can provide physiological homeostasis via regulating specific host signaling pathways. Future studies are needed using different animal models in order to clearly elucidate the key concepts to understand the relationships between gut microbiota and human health and microbiota-related diseases. 

The onset and development of tumor pathologies seem to have a direct relationship with our microbiota, although the molecular mechanisms are still to be defined [104]. It is known that some metabolites of bacterial origin [105] are able to trigger pro- or anti-cancer pathways: for example, some SCFA—such as butyrate—can have an action of this type in some cell lines (adenocarcinoma cells), altering the cell cycle and ultimately leading to cell apoptosis [106,107]. In breast cancer, gut microbiota-derivate metabolites are important in modulating cancer cell function and in creating and maintaining the tumor microenvironment [108].

Clearly, the diet is a prominent source of these metabolites; for example, high-fat and high-protein diets are a feature of the modern Western diet [109,110], which is one of the risk factors for the occurrence of cancer [111,112]. It therefore seems correct to hypothesize that a diet rich in foods that promote the metabolism of SCFA producing bacteria may somehow contribute to the onset of neoplastic diseases in some subjects. The good news is that lifestyle, diet and physical activity clearly outweigh whatever genetics we have inherited in shaping the composition, structure and function of the gut microbiota.

The goal for the future is to utilize a precision medicine: the possibility to personalize microbial replacement therapies for each patient’s conditions, thus making the intervention as effective as possible. A recent study by Elinav and colleagues [99] suggests that probiotics do not have the same (or reproducible) effect in human patients. The collected samples of microbiomes—after four weeks supplements integration—showed that the beneficial bacteria were found in the digestive tracts of only some of the people. In the others, the bacteria were present only in stool samples, not in their digestive tracts [113]. 

In the past decades, bariatric surgery has been the choice of election to extreme situations of weight gain; modifying the anatomy of the gastrointestinal system, the intervention modulates nutrient transit and impacts upon the gut microbiota state [114].

**Figure 3:**
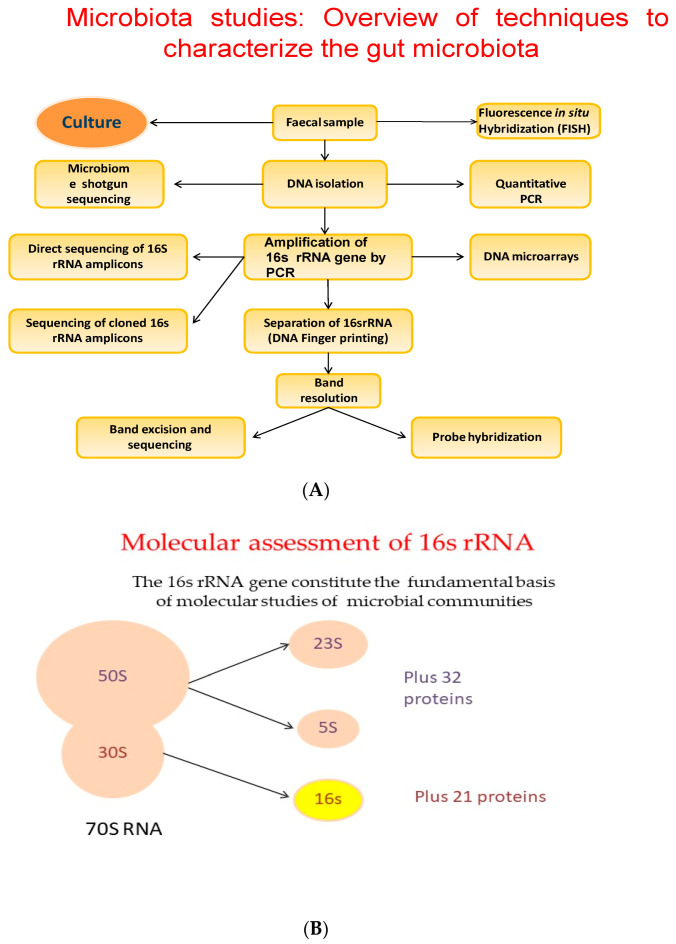
(**A**) Overview of techniques to characterize the gut microbiota. (**B**) The 16 s rRNA gene constitutes the fundamental basis of the molecular studies of microbial communities.

For a long time, FMT has been seen as an extremely promising technique. Actually, it is the privileged technique for *Clostridium* difficile infection treatment [115]. There may be the possibility to choose the most appropriate donor–recipient matching for an FMT intervention. Recently, FMT was also suggested as a possible tool in cancer management as well as cancer-treatment associated complications. Preliminary reports show that FMT could stimulate the cancer immunotherapy effect, significantly improving the patient’s clinical prognosis [116]. The progress of our knowledge on the microbiota and its relationships with our metabolism, will allow us to hypothesize therapies that will be increasingly optimized according to the individual person and his personal characteristics, thus choosing the most suitable bacterial strains to improve the patient intestinal ecosystem, with greater benefits for its metabolism. Today, new techniques such as shotgun metagenomics, metatranscriptomics and metaproteomics, and bioinformatics tools have helped us to identify bacterial communities, highlighting the differences in healthy versus obese microbiota. Metabolomics, the study of the non-protein small molecules that include products of metabolism, could not only help us to identify which species are there, but also to elucidate the metabolic relationships between species and their interactions with the host metabolism [18,117]. The 16 s rRNA gene constitutes the fundamental basis of the molecular studies of microbial communities (Figure 3A,B) [111].

In order to be more effective in the prevention and treatment of specific pathologies, alternative techniques called biotherapeutics are being developed for the use of stool donors. For example, the artificial synthesis of feces—live bacterial metabolites produced in vitro by fermentation, starting from selected bacterial strains—is actually under clinical trial and seems to be very promising [117].

## 8. Discussions

Specific alterations in the human gut microbiota are characteristic in obesity and obesity-related metabolic diseases. Obesity is conditioned by a myriad of factors, and the microbiota is certainly an important player. The analysis of the data obtained from experimental studies allow us to hypothesize that changes in the composition of the microbiota may be the cause, and not simply the consequence, of alterations in human metabolism. If these premises are correct, then the bacterial flora could be an ideal target for possible interventions in the prevention or treatment of diseases related to obesity [118,119,120]. Metagenomics and metabolomics studies could really improve our knowledge, facilitating microbiota manipulation as clinical therapy. 

Modulation of the gut microbiota could improve metabolic parameters in humans, as suggested by several dietary intervention studies, but a causal role of the gut microbiota in such experiments has not been established. As previously described, there are many variables that can profoundly alter the outcome of personalized nutritional strategies: finding the real correlation between a dietary intervention and a metabolic change is still a goal for scientists to achieve. 

Clinical trials that investigate the role of diet-induced modulation of the gut microbiota on host metabolism are needed; those analysis will help us to understand the interactions at molecular level for the observed correlations between our metabolism and microbiota changes, clarifying whether dietary modulation of the gut microbiota can really induce metabolic changes in the host metabolism, thus improving human health [121,122,123,124,125,126,127,128,129]. However, it should be emphasized that “health-associated” microbiota can be present in various forms, such as structure, composition and functionality; therefore, although there are common characteristics, each of us will have his own personal microbiome, suited to his specific needs and characteristics. 

## 9. Conclusions

Overweight and obesity have become very serious problems worldwide, also due to their repercussions on the health system; it therefore becomes of paramount importance to develop strategies that can help to limit the damage.

The microbiota is certainly deeply involved in this situation: the analysis of the bacterial flora is an important indicator for assessing or own personal metabolism, but above all, it can be an ideal target to improve individual health. The use of prebiotics and probiotics must certainly be part of the global strategy to counter obesity, considering the benefits and the almost total absence of risks in their use.

New emerging techniques, or the improvement of existing procedures—such as FMT—could be of paramount importance to help weight loss and/or improve the clinical state in obese people: in the future the scientific advancement could allow that microbiota recovery treatments may become a common choice in the treatment of patients with diseases related to obesity and metabolic dysbiosis in general.

## Figures and Tables

**Table 1:** Major bacteria phyla and genera predominating in human gut microbiota.

Phyla	Representative Genera
**Firmicutes (60–80%)**	*Ruminococcus*
	*Clostridium*
	*Lactobacillus*
	*Enterococcus*
**Bacteroidetes (20–30%)**	*Bacteroides*
	*Prevotella*
	*Xylanibacter*
**Actinobacteria (<10%)**	*Bifidobacterium*
**Proteobacteria (<1%)**	*Escherichia*
	*Enterobacteriaceae*

**Table 2:** Overview of some dietary intervention and microbiota modification. Animal-based diet: composed of meats, eggs, and cheeses; plant-based diet: rich in grains, legumes, fruits, and vegetables.

Dietary Intervention	Duration	Microbiota Modification	Features	Reference
**Animal-based diet**	5 days	*↑* *Alistipes, Bilophila, Bacteroides;* *↓* *Roseburia, Eubacterium rectale, Ruminococcus bromii*	No effect reported	[73]
**Plant-based diet**	5 days	*↑* *Roseburia, Eubacterium rectale,* *Faecalibacterium prausnitzii* *↓* *Prevotella*	No effect reported	[73]
**Macrobiotic diet**	3 weeks	*↑* *Ruminococcus, Blautia Akkermansia, Faecalibacterium,*	↓ postprandial glucose, LDL, insulin resistance	[74]
**Whole grain diet**	3 weeks	*↑* *Bifidobacterium, Lactobacillus*	No effect reported	[75]
**Hypocaloric high protein diet**	6 weeks	*↑* *Diversity (not specified)*	↓ insulin resistance, triglycerides	[76]
**Western style diet**	4 weeks	*↑* *Bifidobacterium,* *↓ Bacteroides, Odoribacter,* *Desulfovibrionaceae,* *Ruminococcus*	No effect reported	[77]
**Mediterranean diet**	12 months	*↑* *Roseburia, Oscillospira,* *↓ Prevotella*	↑ insulin sensitivity	[78]
**Low-fat, high-carbs diet** **(LFHCD)**	12 months	*↑* *Prevotella, Faecalibacterium prausnitzii,* *↓ Roseburia*	↑ insulin sensitivity	[78]
**Very-low calorie diet**	3 months	*↓* *Akkermansia, Alistipes, Clostridium leptum,* *↓* *Bacteroides*	↓ insulin resistance	[79]
**Strict vegetarian diet**	6 weeks	*↓* *Firmicutes/Bacteroidetes, Enterobacteriaceae,* *↑* *Bacteroides fragilis, Clostridium*		[80]
**Galacto-oligosaccharide supplement**	6 weeks	*↓* *Blautia hydrogenotrophica,* *↓* *Enterorhabdus, Slackia, Howardella, Clostridia, Streptococcaceae, Subdoligranulum*	No effect reported	[81]
**Whole grain–enriched diet**	8 weeks	*↑ Faecalibacterium prausnitzii* *Prevotella copri*	↓ inflammatory markers	[82]
**Refined grain diet**	8 weeks	*↑ Bacteroides thetaiotaomicron*	No effect reported	[82]

**Table 3:** Dietary interventions with representative prebiotics and probiotics.

Prebiotic/Probiotic Strain	Recommended Daily Dose Colony-Forming Units (CFUs)
*E. faecium* LAB SF68	10^8^ CFUs
*S. boulardii*, from *S. cerevisiae*	1 g or 4 × 10^9^ CFUs
*L. rhamnosus GG*	10^10^–10^11^ CFUs
*L. casei* DN-114 001 from fermented milk	10^10^ CFUs
*Bacillus clausii*	2 × 10^9^ spore
*L. acidophilus* CL1285 *+ L. casei* LBC80R	5 × 10^10^ CFUs
Inulin/Fructooligosaccharides (FOS)	15 g
Inulin/FOS + *B. longum* 2 × 10^11^/*die*	6 g
